# The Use of Flocculation as a Preconcentration Step in the Microalgae Harvesting Process

**DOI:** 10.1111/ppl.70366

**Published:** 2025-06-27

**Authors:** Zivan Gojkovic, Aleksandra Skrobonja, Vuk Radojicic, Benedetta Mattei

**Affiliations:** ^1^ Department of Life, Health and Environmental Sciences University of L'Aquila L'Aquila Italy; ^2^ Department of Physical Chemistry Vinča Institute of Nuclear Sciences, University of Belgrade Belgrade Serbia

**Keywords:** biological flocculants, biomass recovery, chemical flocculants, microalgae harvesting, pilot‐scale flocculation

## Abstract

Flocculation is a widely utilized separation technique in colloid chemistry, the chemical industry, and wastewater treatment. However, its application in microalgal biomass production and downstream processing remains limited. Biomass harvesting represents a significant cost factor in large‐scale microalgae production, with the separation of biomass from the liquid medium accounting for up to one‐third of total production expenses. The use of flocculants as a pre‐treatment step has emerged as a promising approach to reducing these costs. Laboratory‐scale flocculation is commonly employed to optimize flocculant dosage and assess strain‐specific flocculation efficiencies. Meanwhile, pilot‐scale flocculation, when applied before centrifugation, provides critical insights into the applicability of specific flocculant–strain combinations and their impact on yield and biomass quality. Numerous studies have investigated the flocculation behavior of various microalgal strains using a wide range of chemically distinct flocculants at the laboratory scale. This review highlights recent advancements in microalgal flocculation as a pre‐centrifugation strategy and outlines future perspectives for achieving cost‐effective, large‐scale microalgal biomass production as a globally viable resource.

## 1 Introduction

Mass production of microalgae faces economic challenges that hinder its potential as a major source of global plant‐based biomass. The primary bottlenecks in this process are the harvesting and extraction/cell disruption steps. Centrifugation is a very reliable, commonly used, robust, fast, and effective method for processing most microalgal cultures (Barros et al. 2015; Rawat et al. 2011; Singh and Patidar 2018; Tang et al. 2020). In many cases, centrifugation alone is sufficient as a one‐step biomass separation and dewatering process. However, certain microalgal strains require preconcentration prior to centrifugation (Show and Lee 2014). Despite its advantages, centrifugation has limitations for large‐scale cultivation systems aimed at producing low‐cost bulk biomass. These include high initial investment costs for industrial centrifuges and complementary equipment, significant energy demands, and the risk of cell damage due to the sheer stress, which collectively make centrifugation less suitable for such applications (Tan et al. 2020; Okoro et al. 2019; Singh and Patidar 2018; Tang et al. 2020). Furthermore, achieving maximal harvesting efficiency with centrifugation is not cost‐effective (Dassey and Theegala 2013). High efficiencies require longer retention times in the centrifuge bowl, primarily due to the small size of microalgal cells (Barros et al. 2015). For instance, single‐step centrifugation requires approximately 2 kWh (7.2 MJ) of electrical energy (Fasaei et al. 2018) to process 1.0 m^3^ of microalgal culture with a biomass concentration of 1 g_DW_/L. Given that the energy content per gram of biomass is about 20 kJ/g_DW_ based on its average combustion enthalpy (Tredici 2010), the centrifugation step alone already consumes approximately 36% of the total energy stored in the biomass. To make large‐scale microalgal production economically viable, alternative harvesting processes must be considered. Several intrinsic characteristics of microalgal cultures complicate cost‐effective harvesting. These include small cell sizes (1–30 μm in diameter), colloidal stability in suspension due to the negative surface charge of the cells (Gojkovic et al. 2020; Spain and Funk 2024), and low biomass concentrations in the harvested culture medium (0.2–5.0 g_DW_ or 0.02%–0.5% dry solids) (Haver and Nayar 2017; Milledge and Heaven 2012; Sanyano et al. 2013; Singh and Patidar 2018; Trovão et al. 2019). Alternatives to centrifugation that may be applied to large‐scale cultures include filtration, flotation, electrocoagulation, and gravity sedimentation (Lucakova et al. 2021; Milledge and Heaven 2012; Salim et al. 2012; Sanyano et al. 2013). Among these, flocculation is widely recognized as a promising alternative for microalgal harvesting. It is commonly used in the chemical industry for water treatment and in the food industry to separate colloidal substances, and it has been identified as a viable option for microalgae harvesting (Li et al. 2020). The addition of flocculants in large‐scale microalgal production also enhances biomass sedimentation while minimizing biomass contamination, enabling the reuse of used culture medium and reducing the environmental impact (Barros et al. 2015; Granados et al. 2012; Grima et al. 2003). Bioflocculation refers to the aggregation of microalgal cells induced by the introduction of microbial agents that act as natural flocculants (Singh and Patidar 2018).

Flocculation has been extensively studied at the laboratory scale with various flocculants across a wide range of microalgal strains, including *Chlorella sorokiniana* (Figueira Garcia et al. 2024; Xu et al. 2013), *Chlorella vulgaris* (Hadjoudja et al. 2010; Matho et al. 2019; Oh et al. 2001; Salim et al. 2011; Spain and Funk 2024; Vu et al. 2021), *Chlorella minutissima* (Papazi et al. 2010), *Chlorella protothecoides* (Letelier‐Gordo et al. 2014), *Chlorella zofingiensis* (Zhang et al. 2012), *Parachlorella kessleri* (Vandamme et al. 2010), *Chlamydomonas reinhardtii* (Gerde et al. 2014), *Dunaliella tertiolecta* (Figueira Garcia et al. 2024), *Dunaliella salina* (Besson et al. 2019), *Desmodesmus brasiliensis* (Ndikubwimana et al. 2016), *Scenedesmus dimorphus* (Hansel et al. 2014), *Scenedesmus obliquus* (Kim et al. 2018; Koley et al. 2017; Salim et al. 2011), *Scenedesmus ellipsoideus* (Úbeda et al. 2017), *Scenedesmus* sp. (Gerde et al. 2014; Kumar et al. 2021; Matter et al. 2016; Selesu et al. 2016), *Tetraselmis striata* (Figueira Garcia et al. 2024), *Tetraselmis suecica* (Knuckey et al. 2006), *Thalasiosira pseudonana* (Knuckey et al. 2006), *Phaeodactylum tricornutum* (Şirin et al. 2012; Vandamme et al. 2015), *Neochloris oleoabundans* (Beach et al. 2012; Salim et al. 2011), *Nannochloropsis* sp. (Shen et al. 2013), *Muriellopsis* sp. (Granados et al. 2012), *Scotelliopsis reticulata* (Spain and Funk 2024), *Schizochytrium limacinum* (Gerde et al. 2014), *Botryococcus braunii* (Kim et al. 2013), *Botryococcus* sp. (Rahul et al. 2015), *Euglena gracilis* (Zhu et al. 2025), *Microcystis aeruginosa* (Hadjoudja et al. 2010; Nie et al. 2023; Wang et al. 2013). Despite the extensive laboratory‐scale investigations, pilot‐scale studies involving larger volumes in the operational environments remain limited. These studies are crucial for assessing the feasibility of flocculation as a preconcentration step in large‐scale biomass production (Besson et al. 2019; Figueira Garcia et al. 2024; Knuckey et al. 2006; Koley et al. 2017; Selesu et al. 2016). This review tends to summarize recent advances in microalgal flocculation prior to centrifugation on lab‐scale and pilot‐scale. We also propose some future prospectives in the effort to achieve economically viable large‐scale production of microalgal biomass as a global commodity.

## 2 Flocculation Process in Microalgal Cultures

### 2.1 Stability of Microalgal Cells in Aquatic Environments

When cultivation is completed and the microalgal culture is transferred from the photobioreactor (PBR) to a tank, the absence of culture mixing, pumping or air bubbling, combined with natural gravity, can result in spontaneous biomass settling, a process known as autoflocculation (Singh and Patidar 2018). Additionally, as CO_2_ addition stops, the pH of the culture medium increases, potentially causing the simultaneous precipitation of carbonate salts and microalgal cells. This occurs under elevated pH conditions, where carbonate becomes the predominant form of dissolved carbon (Şirin et al. 2012). Autoflocculation is a simple, natural, chemical−free process governed by Stoke's law (Mathimani and Mallick 2018; Moran 2018):

(1) $$V=\frac{2\;\left(\rho_{\text{cell}}-\rho_{\text{medium}}\right)g\cdot R^{2}}{9\eta }$$

where *V* (m s^−1^) is the particle settling velocity, *R* (m) is the cell radius, *g* is the gravitational acceleration (9.81 m s^−2^), *η* is the dynamic viscosity of the medium (kg m^−1^ s^−1^), and *ρ*_cell_ and *ρ*_medium_ represent the densities of the cell and culture medium (kg m^−3^), respectively.

From this equation, it can be observed that the autosettling velocity of microalgae primarily depends on cell size ($R^{2}$), which implies that for most species, this velocity is very low due to the small size of microalgal cells (1–30 μm in diameter) (Gojkovic et al. 2020; Spain and Funk 2024). It should be noted that this equation is not directly applicable to motile flagellated microalgae such as *Chlamydomonas* sp. Additionally, the density of microalgal biomass is very close to that of water, which further limits settling velocity. Using Stoke's law, the settling velocity of *Chlorella* sp. cells can be calculated, assuming *ρ*_cell_ = 1030–1100 kg m^−3^ (Mathimani and Mallick 2018), an average cell radius 2–4 μm (Gojkovic et al. 2020) and approximating the diluted culture medium with water at 25°C (*ρ*_medium_ = 997 kg m^−3^, *η* = 0.89 × 10^−3^ kg m^−1^ s^−1^). For *R* = 4 μm and *ρ*_cell_ = 1100 kg m^−3^, *V* is calculated to be 4.04 μm s^−1^. This indicates that a single *Chlorella* sp. cell (of 4 μm radius) would take nearly 6 days to sediment in a 2 m water column. This slow sedimentation rate suggests that autosettling cannot be solely relied upon as a preconcentration step before centrifugation of microalgal mass cultures. The process is time‐consuming, uneven and suitable only for species that readily autoflocculate (Yang et al. 2021). In general, all microalgal species that remain stable in the culture medium after cultivation or after mixing/bubbling ceases are considered autoflocculating species, such as the majority of *Chlorella* strains (Salim et al. 2011, 2012; Spain and Funk 2024). Species that readily flocculate even in the PBRs during the late stationary phase are considered flocculating species. From an industrial perspective, the use of flocculants is necessary to preconcentrate cultures, even for autoflocculating microalgae, to ensure process efficiency.

### 2.2 Impact of Cell Wall Composition and Structure on Surface Properties and Flocculation Behaviour of Microalgae

The composition of the microalgal cell wall influences cell surface charge, hydrophobicity, and interactions with surrounding particles. Microalgal cell walls are primarily composed of hydrophilic macromolecular composites, including semicrystalline cellulose and matrix‐phase polysaccharides, but can also contain proteins and lipids, with varying structural complexity, and their composition and structure vary greatly among species. Green algae, such as Chlorella, have cell walls rich in cellulose and polysaccharide composites (Fuertes‐Rabanal et al. 2024; Weber et al. 2022), while, for example, *H. pluvialis* and Scenedesmus sp. have lower amounts of polysaccharides at their surface and higher quantities of proteins. Notably, the surface of Scenedesmus sp. contained significantly more proteins than other compounds, along with a higher proportion of lipids than carbohydrates (Spain and Funk 2022). Diatoms, on the other hand, possess silica‐based frustules accompanied by organic components that are diverse in chemical nature and distribution (Yang et al. 2023). Despite many research reports, the detailed composition remains only partially understood due to its complexity and the technical expertise required for further investigation. The presence of negatively charged functional groups, such as carboxyl and sulfate groups, contributes to the overall negative surface charge of the cells, which can affect electrostatic interactions and hinder spontaneous aggregation. In contrast, non‐polar groups on the cell wall or extracellular matrix of microalgae, such as hydrophobic amino acids in proteins or polymers containing non‐polar groups, play a key role in increasing cell surface hydrophobicity and reducing wettability (Ndikubwimana et al. 2015). These hydrophobic components create a barrier that limits water interaction, favouring cell aggregation and enhancing flocculation potential. The degree of hydrophobicity can vary between species depending on the composition and distribution of these groups.

Additionally, environmental factors such as nutrient availability and growth phase can influence the expression of hydrophobic surface groups, further affecting the colloidal stability and aggregation behavior of microalgal cells. The balance between hydrophobic and hydrophilic groups affects the microalgae's colloidal stability, adhesion properties, and flocculation behavior. Understanding the contribution of surface groups is essential for optimizing biomass harvesting methods and improving microalgal recovery in large‐scale production systems.

Under certain environmental conditions, such as changes in pH or ionic strength, cell surface charge can be neutralized, promoting cell aggregation through van der Waals forces or hydrogen bonding. Additionally, the secretion of extracellular polymeric substances (EPS) by some microalgal species (e.g., *Scotelliopsis reticulata* (Spain and Funk 2024) and Chlorococcum sp. (Lv et al. 2018)) enhances flocculation by forming a sticky matrix that facilitates cell adhesion (Zhou et al. 2024).

### 2.3 Electric Double Layer and Electrokinetic Zeta (ζ) Potential as a Measure of the Cell Stability in Liquid Media

**FIGURE 1:**
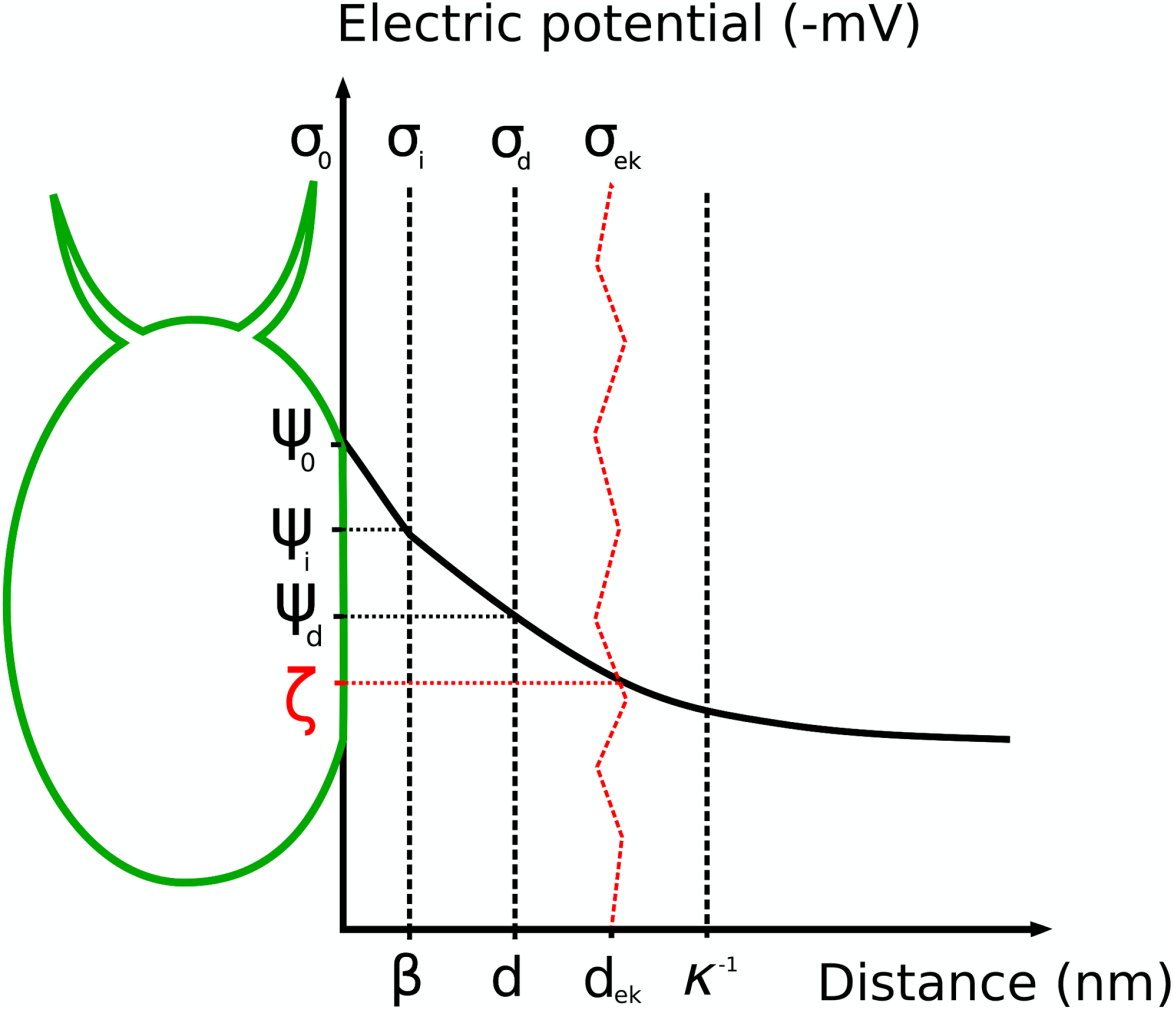
Schematic representation of the electrical double layer surrounding a microalgal cell in the culture medium, illustrating charge densities and potentials. ψ_0_ represents the cell surface potential; ψ_i_ is the potential at the inner Helmholtz plane, located at distance *β* from the cell surface; ψ_d_ denotes the Stern potential; ζ (shown in red) is the electrokinetic or zeta potential, located at the shear plane (assumed to be at approximately *d*_ek_ ≈ 0.5 nm from the cell surface (Goodwin 2009)); *κ* ^−1^ represents the thickness of the electrical double layer or Debye length, while *d* is the thickness of the Stern layer. Charge densities are denoted as follows: *σ*_0_ is the cell surface charge density, *σ*_i_ is the charge density at the inner Helmholtz layer, *σ*_d_ is the charge density at the diffuse layer and *σ*_ek_ is the charge density at the shear plane, corresponding to the electrokinetic zeta potential (ζ). For negatively charged microalgal cells, all these potentials are negative, and the *y*‐axis ranges from 0 to −50 mV. Adapted from Delgado et al. (2007) and Kontogeorgis and Kiil (2016a).

Why are some microalgal species flocculating while others are not? Why are polyvalent cationic substances able to flocculate microalgae? The answer to these questions lies partially in the natural negative charge of microalgal cells, which behave as colloid particles in a liquid medium. Similar to colloidal particles, microalgal cells are surrounded by an electrical double layer that determines their charge. This electrical double‐layer consists of two sublayers: (1) the Stern layer, which is adsorbed onto the cell surface and (2) the diffuse layer that surrounds it (Kontogeorgis and Kiil 2016a). The Stern layer is further subdivided into an inner Helmholtz layer, which is in direct contact with the cell surface up to the inner Helmholtz plane and the outer Helmholtz layer, located between the inner Helmholtz layer and the outer Helmholtz plane (Delgado et al. 2007). The thickness of the Stern layer (*d*) is only a few angstroms, while the total thickness of the double layer, known as the Debye length (*κ* ^−1^), depends on factors such as the type of medium, temperature and ionic strength of the solution (Kontogeorgis and Kiil 2016a). Each surface within the electrical double layer has its own potential (measured in mV): the surface potential (ψ_0_), the potential at the inner Helmholtz plane (ψ_i_), located at a distance *β* (0 ≤ *β* ≤ *κ* ^−1^) from the surface, and the potential at the Stern layer (ψ_d_). Each surface also has a corresponding surface charge density (*σ*): *σ*_0_—represents the cell surface charge density, *σ*_i_ − charge density at the inner Helmholtz layer, *σ*_d_ − charge density in the diffuse layer. Due to electroneutrality, the net charge density of the system is *σ*_0_ + *σ*_i_ + *σ*_d_ = 0. The surface charge densities of *C. vulgaris* (average cell surface area = 55 μm^2^) and *M. aeruginosa* (average cell surface area = 95 μm^2^) are determined to be 1.1 × 10^−5^ neq/cell and 1.9 × 10^−5^ neq/cell, respectively (Henderson et al. 2008a). The separation of charges in the electric double layer between the Stern and diffuse layers generates an electric potential (Matho et al. 2019), known as the electrokinetic or zeta (ζ) potential. The zeta potential is defined as the electric potential at the shear plane of the particle (Figure 1). The shear plane is located close to the diffuse layer (Delgado et al. 2007), approximately 0.5 nm from the cell surface (Goodwin 2009), where the first layer of solvated ions moves relative to the surface and part of the diffuse layer is sheared off by particle motion (Kontogeorgis and Kiil 2016a; Matho et al. 2019). The electric double layer moves along with the particle, and the zeta potential reflects the potential at this shear surface (Delgado et al. 2007). For aqueous solutions at 25°C, the Debye length can be calculated using the simplified equation:

(2) $$\kappa^{-1}\left(\mathrm{nm}\right)=\frac{\left(0.429\;\mathrm{nm}\;{\left(\mathrm{mol}\;L^{-1}\right)}^{\frac{1}{2}}\right)}{\sqrt{2\cdot I}}$$

where I is the ionic strength of the solution (Kontogeorgis and Kiil 2016a).

The zeta potential (ζ), defined at the surface of shear, is the lowest (in absolute value) of the four potentials in the electrical double layer, but is the only parameter measurable through the electrophoretic mobility (EM) of the cells. In practice, the cell surface potential is approximated by the zeta potential, as the difference between these two potentials is negligible (Kontogeorgis and Kiil 2016a). EM (in m^2^ V^−1^ s^−1^) is calculated as EM = *u*·*E* ^−1^ where u (in m s^−1^) is the velocity of the charged particle (e.g., a microalgal cell) and *E* (in V m^−1^) is the electric field strength (Kontogeorgis and Kiil 2016a). Zeta potential is then derived using the Smoluchowski equation (Henderson et al. 2008a):

(3) $$\zeta =\frac{\mathrm{EM}\cdot \eta }{\varepsilon \varepsilon_{0}}$$

where *η* is the dynamic viscosity of the medium (kg m^−1^ s^−1^), ε is the relative electric permittivity, and *ε*_0_ is the vacuum permittivity (8.854 × 10^−12^ C^2^ J^−1^ m^−1^). This equation is applicable for larger particles, such as microalgae, under conditions where the Debye length (*κ* ^−1^) is much smaller than the particle radius (*κ*·*a* > > 1, where *κ* is the Debye–Hückel parameter or inverse double layer thickness), and the double layer is thin (Delgado et al. 2007; Henderson et al. 2008a; Matho et al. 2019). The zeta potential provides an apparent measure of the surface charge of microalgal cells (Henderson et al. 2008a). The isoelectric point (IEP) is defined as the pH of the medium at which the electrostatic double layer is neutralized (ζ = 0 mV, with sufficiently high [H^+^] concentration), leading to system destabilization and flocculation. At the IEP, repulsive electrostatic forces are minimized, and attractive van der Waals forces dominate, causing cell aggregation (Henderson et al. 2008a). Charge neutralization is an important mechanism for the flocculation of microalgae even at neutral pH (Henderson et al. 2008a). An increase in salt concentration in the medium negatively affects cell stability by (1) reducing the zeta potential and (2) compressing the double layer, resulting in a decreased Debye length (κ^−1^) (Kontogeorgis and Kiil 2016a). To minimize such effects, zeta potential measurements are typically conducted in deionized water or low ionic strength solutions (Gojkovic et al. 2020). A stable colloidal suspension is usually associated with a zeta potential of at least ±30 mV (Kontogeorgis and Kiil 2016a), which aligns with experimental values reported for microalgae. For example, the zeta potential of *Botryococcus* sp. was ζ = −25.5 mV, increasing to +3.49 mV after treatment with cationic inulin, demonstrating the flocculant's efficiency (Rahul et al. 2015). Similarly, *S. limacinum* (ζ = −9.97 mV), *C. reinhardtii* (ζ = −19.95 mV) and *Scenedesmus* sp. (ζ = −20.60 mV) show varying tendencies for flocculation, with *S. limacinum* being the most readily flocculating species (Gerde et al. 2014). For *Scenedesmus* sp., both zeta potential and recovery efficiency (RE, %) increased over a 15‐day cultivation period: ζ = −21.3 ± 1.2 mV, RE = 10% (day 1), ζ = −14.64 ± 0.87 mV, RE ≥ 75% (day 10) and ζ = −10.4 ± 1.26 mV, RE = 89.31% (day 15), indicating autoflocculation (Kumar et al. 2021). For the cyanobacterium *Microcystis aeruginosa* , the zeta potential steadily decreased with increasing pH, from −15 mV at pH = 5 to −50 mV at pH = 10, indicating a stable negative charge of microalgal cells in this pH range (Wang et al. 2013). Another study reported that *M. aeruginosa* had negative ζ (from −15 to −25 mV) in the physiological pH range (pH = 5 to 8 (Sakarika and Kornaros 2016)) with IEP (ζ = 0) at pH ≈ 2.2 (Hadjoudja et al. 2010). The zeta potential of *C. vulgaris* typically ranged from −20 to −30 mV across a pH decrease from 14 to 4 but increased to positive values (−30 to +5 mV) under very acidic conditions (IEP between pH = 2 and 3) (Prochazkova et al. 2015). Another study reported that *C. vulgaris* had negative ζ (−10 to −20 mV) in the physiological pH range (pH = 5 to 8) with IEP (ζ = 0) at pH ≈ 2.9 (Hadjoudja et al. 2010). Zeta potential in the range of pH = 2–12 was ζ = −20 mV to −10 mV for *Chlorella* sp. and *S. obliquus* , with the only difference that ζ in *S. obliquus* reached zero at pH = 2 (IEP), while it remained at ζ = −10 mV at the same pH in the case of *Chlorella* sp. (Kim et al. 2018). It is obvious that *C. vulgaris* has a very stable negative ζ and that its IEP sometimes cannot be reached even at pH = 2 (Kim et al. 2018). Nevertheless, optimum flocculation of *C. vulgaris* can be achieved (even without complete charge neutralization) through flocculant addition that increases zeta potential to values ζ ≥ −16.8 mV (Henderson et al. 2008a). It has been reported that the initial zeta potential of *Chlorella* sp. was ζ = −20 mV, and it increased to −14 mV after flocculation with 25–45 mg/L ferric sulfate at pH = 3–5, suggesting that the IEP was not achieved in this case (Kim et al. 2017). *C. vulgaris* 13–1 cultivated for 11 days in BG11 medium at neutral pH maintained a stable zeta potential of ζ ≈ −20 mV from day 3 of the cultivation period until the end of the experiment (Spain and Funk 2024). Another study found that the zeta potential for culture collection *C. vulgaris* UTEX000265 decreased from −10.6 ± 0.3 mV to −18 ± 0.4 mV upon the addition of 0.12 g/L of chitosan. Moreover, it was reported that the zeta potential of *C. vulgaris* decreased with culture age during the cultivation period, from ζ = −12.45 ± 0.18 mV (day 3) to −20.24 ± 0.04 mV (day 10) (Ma et al. 2016), which is contradictory to the common opinion that mature cultures of microalgae easily flocculate. The difference between the zeta potential of *C. vulgaris* measured in the culture medium and deionized water was achieved only at pH = 4–7, where ζ in the culture medium was lower (ζ = −30.1 ± 0.6 mV) compared to that of *C. vulgaris* in deionized water (ζ = −22.4 ± 0.45 mV) (Matho et al. 2019). Furthermore, ζ at pH = 1 was −4.29 mV and has not reached IEP in both liquids, even under very acidic conditions (Matho et al. 2019). Authors further reported that, with the addition of chitosan, the zeta potential of microalgal cultures increased from −15 mV at 5 mg/L chitosan, reaching positive values at ≥20 mg/L chitosan. The ζ value further increased in magnitude, even when IEP (ζ = 0) was surpassed. Interestingly, ζ potential of natural cationic starches (corn and potato) used as flocculants was reported to be in the range +15 to +16 mV in pH = 5 to 10, with a slight decrease in the higher pH (Anthony and Sims 2013). A study on different *Chlorella* strains demonstrated that the carbohydrate composition of the algal cell wall significantly influenced the flocculation efficiency with chitosan. Higher cell wall polysaccharide content positively correlated with improved flocculation, particularly at pH 8.5, where hydrogen bonding likely plays a key role (Cheng et al. 2011). On the other hand, the addition of 210 mg/L of the tannin‐based flocculant Tanfloc to *Scenedesmus* sp. culture at pH = 7.8 resulted in a small positive ζ potential (+0.27 ± 2.43 mV), which was confirmed by the high flocculation of the culture (RE = 97% ± 1%) (Selesu et al. 2016). The wide variation in reported zeta potential values for the same microalgal species highlights the importance of strict control over pH, temperature, and ionic strength in electrokinetic studies (Delgado et al. 2007). Uniform protocols are essential to ensure reproducibility and reliable interpretation of zeta potential measurements for microalgal flocculation.

### 2.4 Mechanisms of Microalgal Flocculation Induced by Positively Charged Flocculants

This review focuses on methods of microalgal flocculation induced by positively charged flocculants. These methods include the use of polyvalent metal cations (e.g., Al^3+^ and Fe^3+^), acid/base flocculation (via the addition or removal of protons, [H^+^]), polyvalent cationic polymers and natural organic flocculants such as starch and chitosan. It is important to distinguish the addition of organic or biological substances from bioflocculation. As stated in the Introduction, bioflocculation refers to microalgal flocculation induced by the addition of microbial material that acts as a flocculant (Singh and Patidar 2018). Examples of bioflocculating agents include yeast (Prochazkova et al. 2015), bacteria (Min et al. 2022; Ndikubwimana et al. 2016), filamentous fungi (Zhou et al. 2013), and biomass of various readily flocculating microalgae species (Kawaroe et al. 2016; Salim et al. 2011, 2012), as well as their derivatives, such as culture medium or exopolysaccharides (EPS) (Lv et al. 2018; Spain and Funk 2024). This environmentally friendly approach has several limitations that hinder its application in large‐scale systems. These include potential contamination of the desired biomass with other microbial species, the need for additional cultivation systems to produce and process the flocculating microorganism, and the distinct differences between these systems and traditional PBRs (Salim et al. 2011). Nevertheless, commensal interactions between microalgae and bacteria hold significant potential in various fields of application, including detoxification, bioproduction and wastewater treatment. For instance, such interactions have been demonstrated in consortia involving *Chlamydomonas* sp. with its associated bacterial communities (Torres et al. 2024) and *Chlorella vulgaris* with naturally occurring bacteria *Rhizobium* sp. (Ferro et al. 2019). Furthermore, comparing the efficiencies and impacts of studies utilizing bioflocculation as a biomass pretreatment is challenging due to significant differences in flocculating agents/species, application conditions and possible micro‐contaminations that may affect results but are often overlooked or unreported. For these reasons, bioflocculation is considered less reliable than chemical or cationic natural polymer flocculation and will be briefly mentioned, but not discussed in detail.

Upon the addition of a flocculant, the negative surface charge of the microalgal cells is gradually neutralized, leading to flocculation through one of three modes: patching, bridging, or sweeping (Mathimani and Mallick 2018). Patching occurs when a positively charged flocculant “patches”—adsorbs completely onto the surface of algal cell, being too short to bind other cells as well, thus reversing the local negative charge and attracting opposite charges, which leads to cell sedimentation (Fast et al. 2014; Salim et al. 2011). In bridging, the flocculant binds simultaneously to the multiple algal cells, forming a “bridge” between them (Vandamme et al. 2013). Patching and bridging are considered the primary mechanisms in cationic polymer flocculation, where negatively charged microalgal cells interact with the large positively charged polymer chains, creating regions of opposite charge that promote the simultaneous occurrence of patching and bridging (Yang et al. 2021). In case of chitosan, a long‐chain polymer with multiple cationic groups, bridging occurs when the polymer binds partially to the microalgal cell, leaving unbound charged segments that interact with other cells, resulting in a polymer/microalgal network that precipitates (Fast et al. 2014). Patching occurs when the chitosan molecule fully binds to the microalgal cell, creating a locally positively charged patch that attracts oppositely charged, leading to flocculation (Fast et al. 2014). Choosing cationic polymers with strong affinity for the culture medium and good solubility in water is crucial for achieving complete particle coverage and full adsorption to the cells (Kontogeorgis and Kiil 2016b). Chitosan flocculation includes not only bridging (Rashid et al. 2013), but also sweeping, and charge neutralization, and it is particularly effective under low pH conditions due to its linear molecular structure with positively charged NH_3_^+^ and NH_2_^+^ groups that neutralize the microalgal cell charge (Figueira Garcia et al. 2024; Matter et al. 2019). Bridging and netting are recognized as the main mechanisms in flocculation using chitosan, γ‐glutamic acid, modified larch tannin, cationic inulin, sodium alginate and cationic starch (Bayat Tork et al. 2017; Ma et al. 2016; Matter et al. 2018; Rahul et al. 2015; Rashid et al. 2013; Wang et al. 2013). Cationic starch flocculates microalgal cultures via interparticle bridging and patching as larger polymer structures (tails and loops) adsorb to surrounding cells, resulting in the physical linking of particles, promoting further collision and flocs forming (Hansel et al. 2014). It has been reported that the efficiency of cationic polymer flocculation is highly dependent on dosage, where low doses result in weak bridging and loose flocs, while excessive doses can reduce bridging efficiency due to electrostatic repulsion caused by the accumulation of positive charges (Singh and Patidar 2018; Yang et al. 2021). Excess of cationic polymers usually has an adverse effect via excessive adsorption of positively charged particles to the algal cells, resulting in unfavorable electrostatic repulsion of strong positive charges, which leads to restabilization of the formed flocs (Hansel et al. 2014). Cationic polymers with higher molecular weights are generally more efficient flocculants due to their enhanced bridging capacity (Koley et al. 2017). Sweeping typically occurs when a high concentration of an effective flocculant is introduced, rapidly shifting the flocculation mechanism from bridging to sweeping. This transition leads to the entrapment of microalgal cells within a densely precipitating flocculant network (Gerde et al. 2014; Kim et al. 2017; Salim et al. 2011). High sweeping power has also been observed with the combined effects of high pH and the addition of industrial polyelectrolytes (Knuckey et al. 2006).

Free polyvalent metal cations (Al^3+^, Fe^3+^, Mg^2+^, and Zn^2+^) effectively neutralize the negative charges on the surface of algal cells, promoting cell aggregation and floc formation (Li et al. 2020). To approximately determine the initial metal salt dose depending on the optical density of the microalgal culture, we can use the following equation (Papazi et al. 2010):

(4) $$\text{Flocdose}\;\left(g/L\right)=0.2083\cdot {\mathrm{OD}}_{750}$$

where Floc_dose_ is flocculant dose (g/L). It applies to OD (750 nm) range 0.6–2.4, metal salts: AlCl_3_, Fe_2_(SO_4_)_3_, ZnCl_2_ and was determined for *C. minutissima* cultures (Papazi et al. 2010).

The effectiveness of these polyvalent cations depends on their solubility and electronegativity; more electronegative ions tend to be more efficient flocculants (Singh and Patidar 2018). The electronegativity of common polyvalent cations increases in the following order: Mg < Al < Zn < Fe. It has been reported that electronegativity also influences floc density, with ferric chloride producing denser flocs than aluminum chloride due to its higher electronegativity (Sanyano et al. 2013). Additionally, metal cations hydrolyse in water to form metal hydroxides or react with phosphate to form positively charged ligands, which facilitate flocculation through bridging and patching mechanisms (Kumar et al. 2021; Papazi et al. 2010). Trivalent cations (Al^3+^ and Fe^3+^) are generally more effective than divalent cations (Mg^2+^ and Zn^2+^) (Papazi et al. 2010). Among trivalent cations, Al salts are more efficient than ferric salts due to their higher solubility, higher charge density and lower molecular weight (Barros et al. 2015; Okoro et al. 2019; Papazi et al. 2010). On the other hand, ferric cations are more effective than zinc, as Zn^2+^ has a lower charge density and a higher molecular weight (Mathimani and Mallick 2018; Papazi et al. 2010). The high solubility and charge density of Al^3+^ provide a more extended molecular conformation that helps in bridging between particles and facilitates the neutralization of microalgal cell surfaces (Barros et al. 2015; Mathimani and Mallick 2018; Okoro et al. 2019; Papazi et al. 2010). Ferric cations not only form hydroxides but also bind to phosphate ions and EPS, forming precipitates that effectively trap microalgae in microalgae‐precipitate flocs (Kim et al. 2017).

The addition of ferric sulfate results in the formation of micro‐precipitates attached to microalgal cells (confirmed by optical SEM microscopy images), which then undergo gravitational sedimentation (Kim et al. 2017). However, the use of aluminum, ferric, and zinc salts as flocculants presents several drawbacks. These include potential negative environmental impacts, possible toxicity to algal biomass consumers (e.g., animals and humans), and specific effects such as partial cell lysis caused by aluminum, discoloration of biomass due to ferric salts (yellowish appearance at concentrations > 1 g/L (Papazi et al. 2010)), and the adherence of flocs to the container walls when using zinc salts (Mathimani and Mallick 2018; Udom et al. 2013). For instance, it has been reported that the use of ferric salt and an increase of pH to 11 led to some cell lysis during the flocculation of *S. ellipsoideus* (Úbeda et al. 2017). The addition of more than 90 mg/L polyaluminium chloride or aluminum sulfate resulted in biomass color change in *P. tricornutum* flocs (Şirin et al. 2012). The direct application of polyvalent metal cations for microalgae harvesting involves two key issues: (1) metal ions attach to the obtained biomass, negatively affecting extracted lipids and high‐value chemicals, (2) once used, chemical flocculants are not reusable (Kim et al. 2017). To address these concerns, an acid wash of the flocculated biomass has been proposed to remove metal ions, allowing their recovery for possible reutilization (Kim et al. 2017). Flocculation efficiency of *S. obliquus* did not increase beyond a ferric chloride concentration of 0.2 g/L, suggesting saturation of the solution with polyvalent cations, which may lead to repulsion among larger agglomerates of positively charged biomass flocs with excess adsorbed ferric ions (Koley et al. 2017). Flocculation is also affected by the pH and ionic strength of the culture medium, which are directly related to changes in zeta potential (Branyikova et al. 2018).

Flocculation induced by pH change is governed via two different mechanisms based on the conditions. For acidic precipitation (low pH), the culture flocculates because pH is approaching the IEP of negatively charged microalgal cells, leading to neutralization of the negative surface charge by elevated [H^+^] and subsequent cell flocculation (Letelier‐Gordo et al. 2014). On the other hand, flocculation at high pH is related to the formation of magnesium hydroxide (Mg(OH)_2_) from free Mg^2+^ ions in the medium, which have a large adsorptive surface area and a positive charge able to neutralize the negatively charged cell surfaces, resulting in flocculation (Letelier‐Gordo et al. 2014). Precipitation of Mg begins at pH = 9.5 and completes at pH = 11–11.5 (Şirin et al. 2012). Magnesium hydroxide, with its positive charges, attracts the negative surface charges of the cell and forms a white milky layer coating around the microalgal cells (Koley et al. 2017). It is common that microalgae precipitate at high pH to a certain extent; for example, 68% of *C. protothecoides* biomass readily flocculates at pH = 10 (Letelier‐Gordo et al. 2014). For acid/base flocculation of *C. vulgaris* , *Scenedemus* sp. and *Chlorococcum* sp. by NaOH addition, the optimum pH was approximately 10.6 (Wu et al. 2012). In the *S. obliquus* and *C. vulgaris* culture, pH‐induced flocculation was maximal at pH = 12 with recovery efficiencies of 83.2% and 65.1%, respectively (Koley et al. 2017). Acid‐induced flocculation of *Scenedesmus* sp. and *S. obliquus* required 787.5 and 834.8 mg/L of HNO_3_, respectively, to achieve > 90% recovery (Wu et al. 2015). To achieve the same effect (> 90% recovery), it required 1.4 and 1.0 g/L of NaOH for *Scenedesmus* sp. and *S. obliquus* , respectively (Wu et al. 2015). Flocculation of *P. tricornutum* by increasing pH to alkaline resulted in recoveries of 90% and 98% at pH = 9.8 and pH = 11, respectively (Şirin et al. 2012). The optimal pH for flocculation of *C. vulgaris* and *P. tricornutum* using three different agents varied depending on the flocculant type: pH = 5.5–6.5 for aluminum sulfate, pH = 4–9 for ferric sulfate, and pH = 7–8 for chitosan (Vandamme et al. 2015). Variations in flocculation efficiency across similar studies may be due to differences in growth conditions, specific strain properties (e.g., cell morphology, EPS production) and the predominant cell surface charge (Henderson et al. 2008b; Xu et al. 2013). Marine microalgae species generally require 5 to 10 times higher flocculant doses than freshwater microalgae due to differences in ionic strength and conductivity between freshwater and seawater, which can hamper flocculant activity (Mathimani and Mallick 2018; Uduman, Qi, Danquah, Forde, and Hoadley 2010).

Polyacrylamide‐based organic polymers (such as Magnafloc and Zetag from BASF Chemical Company) are effective flocculants in lower doses than inorganic metal salts, but they may contain acrylamide residues that are potentially carcinogenic and highly toxic to aquatic wildlife, which restrains their applicability in microalgal harvesting (Yang et al. 2021). Two cationic (Magnafloc 155 and 156) and one non‐ionic synthetic polymer (Magnafloc 351) were applied at doses of 2.0, 3.0, and 10 mg/L, respectively, to marine microalgae *Chlorococcum* sp. cultures with recovery efficiencies of 83.9 ± 0.6; 84.5 ± 0.4; and 79.9% ± 0.6% (Uduman, Qi, Danquah, and Hoadley 2010). Authors claimed that microalgal recovery increases with pH, especially for anionic flocculants (unlike in freshwater microalgae), suggesting that higher pH precipitates Ca^2+^ and Mg^2+^ salts in marine microalgal cultures, which improves removal efficiencies (RE) (Uduman, Qi, Danquah, and Hoadley 2010). Ten synthetic polyelectrolytes sold commercially under the name Actipol were tested on freshwater microalgal species: *Muriellopsis* sp., *C. vulgaris* SAG 211–11b, *C. fusca* SAG 211–8b, *S. subspicatus* SAG 86.81, and *Scenedesmus* sp. under the investigation of possible application at large‐scale biomass flocculation for industrial application (Granados et al. 2012). The best‐performing flocculant was Actipol EM16, which, added at 10 mg/L, effectively flocculated > 90% of *Muriellopsis* sp., *C. vulgaris* SAG 211–11b, *C. fusca* SAG 211–8b, *S. subspicatus* SAG 86.81, and *Scenedesmus* sp. with a concentration factor of 26 (Granados et al. 2012). Authors reported that the concentration factor was constant (26) if the flocs were adequately formed at all doses of Actipol flocculants higher than 7 mg/L. Authors concluded that this was due to the properties of the flocs produced, which were the same regardless of the dose of flocculant used (if > 7 mg/L) (Granados et al. 2012). The used culture medium after Actipol flocculation was reused to cultivate *Muriellopsis* sp. with no adverse effect on growth and high final biomass concentration (5.5 g/L), which suggested Actipol has no effect on the photosynthetic efficiency of this species and can be reused (Granados et al. 2012). However, Actipol application to flocculate microalgal biomass destined for human food and animal feed remains uncertain because of possible traces of acrylamide (Yang et al. 2021).

Chitosan, a linear poly‐amino‐saccharide, is a cationic polymer derived from the alkaline deacetylation of chitin. Chitosan is a natural, non‐toxic, highly biodegradable, renewable, and ecologically friendly flocculant and a valuable alternative to metal salts (Rashid et al. 2013; Xu et al. 2013). Chitosan is chemically analogous to cellulose and forms the exoskeletons of crustaceans (e.g., shellfish, shrimp, crabs). It is also the major structural component in insects and fungal cell walls (Nunes and Philipps‐Wiemann 2018). When using chitosan as a flocculant, controlling pH is crucial since its efficiency relies on the protonation of amine groups on its surface, which is most pronounced at low pH (Demir et al. 2020). Protonation of amine groups is insignificant at pH > 10, negatively affecting flocculation (Demir et al. 2020). The isoelectric point of chitosan's poly‐glucosamine macromolecule is pH ≈ 6.5, so the net positive charge responsible for flocculation is more pronounced at acidic pH (Xu et al. 2013). Studies on chitosan‐induced flocculation of the marine microalga *N. oculata* have shown that the mode of action differs in seawater compared to freshwater, with the optimal pH for flocculation being higher in seawater than in freshwater (Blockx et al. 2018). Differences in chitosan‐induced flocculation recoveries, even at similar pH and cell density, may be partially attributed to species‐specific responses of microalgae cultures (Koley et al. 2017). Adding 20 mg/L of chitosan to *C. sorokiniana* had a 99% flocculation efficiency at pH 6 (Xu et al. 2013).

Starch is composed of branched amylopectin and linear amylose, with the latter being more reactive to chemical substitution. Consequently, starches with a higher amylose content typically exhibit a greater degree of substitution (DS) (Anthony and Sims 2013; Peng et al. 2017). Starch modification known as cationic starch is produced from natural starch via esterification that substitutes –OH groups to a certain degree with quaternary ammonium groups in glucose monomer units (Min et al. 2022; Peng et al. 2017). The etherification reaction (through the Williamson ether synthesis mechanism) is performed in the presence of NaOH when OH groups of the anhydroglucose units (AGU) involved in nucleophilic reaction with the quaternary ammonium salt replace H with the amine group (Hansel et al. 2014). Ammonium groups are charged independently from the pH, so they can be applied in a broader pH range than other cationic flocculants (Letelier‐Gordo et al. 2014; Yang et al. 2021). It was suggested that weak alkaline conditions benefit starch flocculation due to the status of quaternary ammonium under high pH (Peng et al. 2017). Experiments on *C. pyrenoidosa* and *B. braunii* cultures resulted in maximal flocculation efficiency of cationic starch at 8 < pH < 9 and much lower flocculation at 4 < pH < 8 and pH = 10 (Peng et al. 2017). Another study reported that cationic starch and two other natural flocculants (Chitosan and Tanfloc) added to *C. vulgaris* and *S. obliquus* were effective at neutral and acidic conditions, but had restricted effectiveness at basic conditions (Yang et al. 2021). These opposite findings can be due to species‐specific differences, different culture media, and experimental setups.

The degree of substitution is the number of substituted groups per monomer unit of the starch (glucose) (Anthony and Sims 2013; Gerde et al. 2014; Hansel et al. 2014). It is calculated from the equation:

(5) $$\mathrm{DS}=\frac{M_{\mathrm{AGU}}\cdot X_{N}}{100\cdot M_{N}-M_{\mathrm{CA}}\cdot X_{N}}$$

where *M*_AGU_ is the molecular weight of anhydrous glucose monomer unit; *M*_N_ is the atomic weight of nitrogen; *X*_N_ is the N content (%) in the modified starch, *M*_CA_ is the molecular weight of the cationizing agent—source of quaternary ammonium moiety—like: glycidyltrimethylammonium unit (GTAC), N‐(3‐chloro‐2‐hydroxypropyl) trimethyl ammonium chloride (CHPTAC), 2,3‐epoxypropyl trimethyl ammonium chloride (EPTAC), 3‐methacryloyl amino propyl trimethyl ammonium chloride (MAPTAC), and so forth. (Anthony and Sims 2013; Hansel et al. 2014; Peng et al. 2017). CHPTAC can also be used for the modification of inulin (Rahul et al. 2015).

The DS is a small unitless number that can range between 0 and 3 (Anthony and Sims 2013). Cationic starches with DS < 0.6 are considered as nontoxic, efficient, cheap, and accessible flocculants for harvesting of the microalgal biomass (Gerde et al. 2014; Vandamme et al. 2010). Cationic starches with low DS are used in paper industry and are approved for contact with food products (Gerde et al. 2014). Cationization of starches is essential for flocculant effectiveness; nevertheless, DS value is not always directly proportional to the RE (%), so more quaternary ammonium groups per starch macromolecule do not necessarily provide faster or more efficient flocculation (Hansel et al. 2014). Flocculation of *S. dimorphus* was stable and uniform using cationic starches with DS range between 0.14 and 0.64, independently on their DS degree (Hansel et al. 2014). The highest RE in that study (> 95%) was achieved with 10 mg/L of cationic starch (Hansel et al. 2014). Zeta potential and indirectly net charge of the cationic starches depend on the adsorption of protons and hydroxyl groups on the surface of the starch particles, as the nitrogen from the quaternary ammonium group attached to the starch molecule can be protonated or deprotonated depending on pH (Anthony and Sims 2013). Potato, corn, and wheat cationic starches with DS of 0.249, 0.183 and 0.178, respectively, were used as flocculants for *C. pyrenoidosa* and *B. braunii* cultures (Peng et al. 2017). Potato, corn and wheat cationic starches added at 91 mg/L to *C. pyrenoidosa* cultures resulted in recoveries of 93.18% ± 1.38%, 96.31% ± 3.24%, and 91.01% ± 1.95%, respectively (Peng et al. 2017). Potato, corn, and wheat cationic starches added at 74 mg/L to *B. braunii* cultures resulted in recoveries of 89.59% ± 1.08%, 93.42% ± 3.27%, and 94.04% ± 2.98%, respectively (Peng et al. 2017). Moreover, there was no difference in efficiency among the three starches because of the plant of their origin, and the flocculation efficiency decreased above the optimal dose, as surplus of positive charge is added to the system (Peng et al. 2017). Cationic starch Greenfloc 120 (commercially used for wastewater treatment and paper mill industry) was applied at 20 mg/L to *P. kessleri* cultures, with RE > 90% (Vandamme et al. 2010). Greenfloc 120 (40 mg/L) applied to *C. protothecoides* cultures was the most effective at pH = 7.7 and pH = 10, with removal efficiency of 95% and 96%, respectively (Letelier‐Gordo et al. 2014). Authors concluded that pH > 10 had no beneficial effects on the cationic starch flocculation efficiency (Letelier‐Gordo et al. 2014). Addition of 45 mg/L cationic starch (with DS = 0.5) had maximal flocculation efficiency (> 85%) in *C. reinhardtii* cultures of *C*_x_ = 0.31 g_DW_/L (Gerde et al. 2014). Cationic starch applied at doses of 40 and 60 mg/L in cultures of *S. obliquus* and *C. vulgaris* , respectively, resulted in recovery > 90% (Yang et al. 2021). Increased doses resulted in decreased recovery for both species, indicating system saturation with positive charge and suggesting patching as the main flocculation mechanism (Yang et al. 2021). Corn and potato cationic starches with DS = 1.34 and 0.82 were tested (doses of 49 to 70 mg/L) for removal of microalgae and suspended solids in wastewater with removal efficiencies of 80% and 60% for corn and potato starch, respectively (Anthony and Sims 2013). Cationic starch induces similar biomass recovery to chitosan under acidic pH; however, under alkaline pH, cationic starch is more effective than chitosan (Min et al. 2022).

Inulin is the general name used for nonfermentable oligo‐ and polysaccharides (β‐D–(2 → 1) linear fructans) naturally found in fruits, that are formed by β‐D–(2 → 1)‐fructofuranosyl moieties with an optional reducing end made by α‐D–(2 → 1)‐glucopyranosyl unit (Pugazhendhi et al. 2019; Rahul et al. 2015; Zhu et al. 2016). Cationic inulin is used as a flocculant for microalgae. It does not change the pH when added to the culture medium and, similarly to chitosan, it has an optimal dose after which adding more flocculant decreases recovery efficiency (Rahul et al. 2015).

Tannins or plant phenols are secondary metabolites of higher plants that are found in almost every part of the higher plants: bark, wood, leaves, fruit, and roots (Wang et al. 2013). Tannins are natural branched anionic polyelectrolytes extracted from the bark and wood of different plants ( *A. mearnsii* , *C. sativa* , *Schinopsis* sp., *L. gmelinii* , etc.) (Min et al. 2022; Selesu et al. 2016; Wang et al. 2013). Tannins are water extracts consisting of flavonoids, hydrocolloid gums and other soluble salts (Selesu et al. 2016). Tannins are chemically modified to acquire a positive charge by the introduction of quaternary ammonium groups (Selesu et al. 2016), similarly to cationic starches. The modification is performed via the Mannich reaction, which involves the introduction of a quaternary nitrogen ion into the tannin's structure (Wang et al. 2013). Flocculation efficiency of the tannin‐based agents is optimal at pH 6 to 8 but decreases as the culture pH rises (Selesu et al. 2016), similar to the behavior observed with chitosan. However, the IEP of natural tannins (pH = 2.0–2.5) is close to the IEP of many microalgal cells, limiting their flocculation efficiency at physiological pH (Wang et al. 2013). On the other hand, IEP of modified larch tannin with introduced active quaternary ammonium group is 8.9 and it is predominantly positively charged in the physiological pH range of microalgae (5.0–8.0 (Sakarika and Kornaros 2016)), making it a very efficient flocculant for microalgae (Wang et al. 2013). Tanfloc is a chemically modified tannin product, produced by introducing amino groups into the tannin structure to improve its flocculation efficiency (Yang et al. 2021).

### 2.5 Effect of Flocculants on Microalgal Physiology

Chlorophyll fluorescence measurements can rapidly indicate the performance of photochemical processes in the photosynthetic apparatus of microalgae (Masojidek et al. 2010). One of the key fluorescence parameters is quantum yield (Q_y_). Q_y_ or maximum photochemical yield of Photosystem II is calculated based on the following equation:

(6) $$Q_{y}=\frac{F_{m}-F_{0}}{F_{m}}$$

where *F*_0_ is minimum or zero fluorescence, *F*_m_ is maximum fluorescence of PSII. *Q*_y_ directly reflects the photosynthetic activity of the culture and indicates the culture's health (Gojkovic et al. 2014; Granados et al. 2012; Vandamme et al. 2015; Yang et al. 2021).

Chlorophyll fluorescence parameters measured in cultures following flocculant addition, or in resuspended biomass post‐flocculation, can provide insights into potential negative effects of the flocculant on microalgal viability and overall culture health. *Q*_y_ value in healthy microalgal cultures is in the range 0.6–0.8, and any toxicity of the flocculants towards microalgae will be rapidly detected and reflected through *Q*_y_ decline (Masojidek et al. 2010). Quantum yield of *C. minutissima* was not affected by the addition of Fe_2_(SO_4_)_3_ (0.75 g/L) or ZnCl_2_ (0.5 g/L) (Papazi et al. 2010) and Mg(OH)_2_ (0.05 g/L) had no noticeable effect on *C. vulgaris* and *P. tricornutum* (Şirin et al. 2012). Based on quantum yield measurements, it was confirmed that application of biological polymers [chitosan, tannin (Tanfloc) and cationic starch] as flocculants did not affect photosynthesis of *C. vulgaris* and *S. obliquus* (Yang et al. 2021). A similar trend was observed for cationic starch Greenfloc 120 added at 20 mg/L to *Parachlorella kessleri*, which had no significant effect on its quantum yield (Vandamme et al. 2010). Similar to chitosan and tannin (Yang et al. 2021), cationic starches of different origin had no adverse effect on quantum yield in algal cultures, even in recultivated cultures inoculated with previously flocculated biomass (Peng et al. 2017). Authors concluded that cationic starches have no immediate or long‐term toxicological effect on microalgal photosynthetic activity and culture health (Peng et al. 2017). The synthetic polymer Actipol had no effect on the photosynthetic efficiency of *Muriellopsis* sp., as the culture's *Q*_y_ was 0.67–0.71, which is equal to the values for healthy microalgae cultures (Granados et al. 2012). Flocculation of *E. gracilis* by the addition of 41.6 mg/L chitosan at pH = 6.49 reduced the *Q*_y_ of the culture by 15.9%, which made authors conclude that cell viability was not compromised (Zhu et al. 2025). Evans blue staining of *Scenedesmus* sp. and *S. obliquus* cells after flocculation with chitosan (1.5 and 17.5 mg/L) and polyacrylamide (10.1 and 75.5 mg/L) indicated that there was no obvious cell lysis and most of the cell walls were intact, so the authors concluded that the cells were not damaged during the flocculation process (Wu et al. 2015). Evans blue staining is a simple and effective method for distinguishing cells with intact cell walls (unstained) from those with compromised cell walls (stained blue). The dye penetrates the protoplasm, selectively coloring cells with disrupted cell walls (Papazi et al. 2010).

### 2.6 Lab‐Scale Flocculation

Lab‐scale flocculation is a widely studied technique in the microalgae harvesting process, in the effort to improve biomass recovery efficiency while reducing energy demands. At the lab‐scale, flocculation studies are essential for optimizing key parameters such as flocculant type, dosage, pH, and mixing conditions, providing critical insights for scaling up to industrial applications.

An overview of summarized efficiency recoveries for different microalgal species using both chemical‐ and natural or synthetic polymer‐based flocculants is presented in below in Tables 1 and 2.

**TABLE 1:** Recovery efficiencies reported in the literature for polyvalent metal salts and chitosan used as microalgal flocculants.

Flocculant	Dose (mg/L)	Recovery (%)	pH	Microalga	References
AlCl_3_ or Al_2_(SO_4_)_3_	152	> 99	6.0	marine *Chlorella* sp.	(Sanyano et al. 2013)
	140	91	> 6.0	*Chlorella*‐like species from wastewater	(Udom et al. 2013)
	250	> 90	7.0	*C. vulgaris*	(Koley et al. 2017)
	250	> 90	7.0	*S. obliquus*	(Koley et al. 2017)
	183.5	> 90	Not controlled	*S. obliquus*	(Wu et al. 2015)
	183.5	> 90	Not controlled	*Scenedesmus* sp.	(Wu et al. 2015)
	1200	85.6	5.84	*Tetraselmis* sp.	(Kwon et al. 2014)
FeCl_3_ or Fe_2_(SO_4_)_3_	143	> 99	8.1	*Chlorella* sp. (marine species)	(Sanyano et al. 2013)
	900	98	3.0	*Chlorella* sp. KR‐1	(Kim et al. 2017)
	150	94.3	7.2	*S. ellipsoideus*	(Úbeda et al. 2017)
	122	93	> 6.0	*Chlorella*‐like species from wastewater	(Udom et al. 2013)
	200	79.2	7.0	*C. vulgaris*	(Koley et al. 2017)
	200	80.2	7.0	*S. obliquus*	(Koley et al. 2017)
	700	92.6	5.98	*Tetraselmis* sp.	(Kwon et al. 2014)
Mg(OH)_2_	24	90	10.7	*C. vulgaris*	(Vandamme et al. 2015)
	1218	73	10.3	*P. tricornutum*	(Vandamme et al. 2015)
ZnSO_4_	750	> 80	Not controlled	*C. minutissima*	(Papazi et al. 2010)
ZnCl_2_	500	> 80	Not controlled	*C. minutissima*	(Papazi et al. 2010)
Chitosan	120	99 ± 0.5	6.0	*C. vulgaris*	(Rashid et al. 2013)
	20	99	6.0	*C. sorokiniana*	(Xu et al. 2013)
	60	52.6 ± 0.5	Not controlled	*C. sorokiniana*	(Figueira Garcia et al. 2024)
	20–40	> 90	7.0	*C. vulgaris*	(Koley et al. 2017)
	20–40	> 90	7.0	*S. obliquus*	(Koley et al. 2017)
	1.5	> 90	Not controlled	*Scenedesmus* sp.	(Wu et al. 2015)
	17.5	> 90	Not controlled	*S. obliquus*	(Wu et al. 2015)
	4000	91.3	7.22	*Tetraselmis* sp.	(Kwon et al. 2014)
	80	73.2 ± 1.1	Not controlled	*T. striata*	(Figueira Garcia et al. 2024)
	150	74 ± 1.2	Not controlled	*D. tertiolecta*	(Figueira Garcia et al. 2024)
	20	90	9.9	*P. tricornutum*	(Şirin et al. 2012)
	46.1 mg/L chitosan + 60 min green light (phototaxis)	93.07	6.49	*E. gracilis*	(Zhu et al. 2025)
	10 + 10 (chitosan) + (Na‐alginate)	> 86	6.0	*S. obliquus*	(Matter et al. 2018)

**TABLE 2:** Recovery efficiencies reported in the literature for different natural and synthetic polymers used as microalgal flocculants.

Flocculant	Dose (mg/L)	Recovery (%)	pH	Microalga	References
Cationic starch	20 (Greenfloc 120)	> 90	5.0–10.0	*P. kessleri*	(Vandamme et al. 2010)
	40 (Greenfloc 120)	98	7.7 and 10.0	*C. protothecoides*	(Letelier‐Gordo et al. 2014)
	60	> 90%	7.0	*C. vulgaris*	(Yang et al. 2021)
	7.1	71.7 ± 7	6.8	*C. vulgaris*	(Bayat Tork et al. 2017)
	40	> 90%	7.0	*S. obliquus*	(Yang et al. 2021)
	45 (DS = 0.5)	> 85%	8.14	*C. reinhardtii*	(Gerde et al. 2014)
	91 (corn cationic starch) (DS = 0.183)	96.31% ± 3.24%	8–9	*C. pyrenoidosa*	(Peng et al. 2017)
	74 (wheat cationic starch) (DS = 0.178)	94.04% ± 2.98%	8–9	*B. braunii*	(Peng et al. 2017)
Other natural polymers	11 Modified tannin	99	7.0	*C. vulgaris*	(Mezzari et al. 2014)
	210 Tanfloc	96.7 ± 1.0	7.8	*Scenedesmus* sp.	(Selesu et al. 2016)
	10 Modified larch tannin	97	7.0–8.0	*M. aeruginosa*	(Wang et al. 2013)
	60 Modified inulin (chicory roots)	88.61	7.4	*Botryococcus* sp.	(Rahul et al. 2015)
	35 Cationic cassia gum	93	7.6	*Chlorella* sp.	(Prochazkova et al. 2015)
	33.15 Modified used yeast	> 90	7–10	*C. vulgaris*	(Prochazkova et al. 2015)
	400 *Moringa oleifera* seed powder	94.5	4–10	*S. obliquus*	(Yang et al. 2021)
	600 *Moringa oleifera* seed powder	95.6	4–10	*C. vulgaris*	(Yang et al. 2021)
Synthetic polymers	10 Actipol EM16	> 90	8.0	*Muriellopsis* sp. *C. vulgaris* *C. fusca*	(Granados et al. 2012)
	0.5 Magnafloc LT25 anionic polymer BASF (Pilot‐scale)	89	10–10.6	*T. pseudonana*	(Knuckey et al. 2006)
	34 Zetag 8819 cationic BASF	98	Not controlled	*Chlorella*‐like species isolated from wastewater	(Udom et al. 2013)
	10 Magnafloc 351 Non‐ionic	79.9	8.0	*Chlorococcum* sp.	(Uduman, Qi, Danquah, and Hoadley 2010)
	10.1 polyacrylamide	> 90	Not controlled	*Scenedesmus* sp.	(Wu et al. 2015)
	75.5 polyacrylamide	> 90	Not controlled	*S. obliquus*	(Wu et al. 2015)
	30 Polyaluminum chloride	80	6.5–7.6	*P. tricornutum*	(Şirin et al. 2012)

Based on the data presented in Table 1, high recovery efficiencies (> 90%) have been observed for several microalgal species at concentrations ranging from 140 and 250 mg/L and within a pH range of 6–7 when Al‐based metal salts were used as flocculants. Similarly, ferric ion‐based metal salts demonstrated recovery efficiencies exceeding 90% at concentrations between 122 and 900 mg/L, across a broader pH range of 3.0–8.1. On average, both aluminum‐ and iron‐based flocculants exhibited comparable recovery efficiencies with reported values of 90.8% ± 4.02% at pH 6.37 ± 0.58 and 90.9% ± 8.02% at pH 6.33 ± 1.64, respectively. Conversely, when chitosan was used as a flocculant, the average recovery efficiency was slightly lower, at 86% ± 12.6%, with an associated pH of 6.95 ± 1.29 and a concentration range of 10–4000 mg/L. These findings indicate that, on average, Al‐ and Fe‐based metal salts yield higher biomass recovery efficiencies compared to chitosan. The most effective Al‐ and Fe‐based flocculants achieved > 99% recovery efficiencies for marine *Chlorella* sp. at concentrations of 152 and 143 mg/L, respectively (Sanyano et al. 2013).

Table 2 compiles literature data on biomass recovery efficiencies achieved using cationic starch and other natural polymers, such as tannins and yeast, as flocculants, in comparison to synthetic polymers. Namely, the average recovery efficiency for cationic starch was calculated to be 89.4% ± 8.25%, with an average pH of 7.79 ± 0.81 and across a concentration range of 7.1 to 91 mg/L. Higher efficiencies, averaging 94.3% ± 7.46%, were reported for various natural polymers at an average pH of 7.46 ± 0.51, with flocculant concentrations spanning from 10 mg/L (modified larch tannin) to 400 mg/L ( *Moringa oleifera* seed powder). Among the natural polymers evaluated, modified tannin exhibited the highest performance, achieving a 99% recovery efficiency for *C. vulgaris* at a concentration of 11 mg/L, (Mezzari et al. 2014), followed by the “Greenfloc 120” cationic starch, with a 98% recovery efficiency for *C. protothecoides* at 40 mg/L (Letelier‐Gordo et al. 2014). In comparison, the use of synthetic polymers resulted in an average recovery efficiency similar to that of cationic starch, with reported values of 88.1% ± 6.36% at an average pH of 8.34 ± 1.38. The flocculant concentrations in this category ranged from 0.5 mg/L (Magnafloc LT25 anionic polymer) to 75.5 mg/L (polyacrylamide). The highest‐performing synthetic polymer was the commercially available “Zetag 8819” cationic polymer, which achieved a 98% recovery efficiency at a concentration of 34 mg/L (Udom et al. 2013).

In general, superior recovery efficiencies were observed with lower doses of cationic starch, synthetic polymers, and chitosan than with metal salt‐based flocculants. However, due to the variability in flocculation efficiency across different flocculants, which depends on factors such as pH, microalgal species, and additional parameters not accounted for in the presented data (e.g., centrifugation conditions), a predictive approximation model is necessary to comprehensively assess lab‐scale flocculation efficiency across a diverse range of microalgal strains.

### 2.7 Pilot‐Scale Flocculation

As previously stated, laboratory‐scale flocculation has been extensively studied across various microalgal species and with different flocculants (see Section 1). In contrast, pilot‐scale studies conducted under operational conditions and aimed at potential applications in large‐scale biomass production are still limited (Table 3) (Besson et al. 2019; Figueira Garcia et al. 2024; Knuckey et al. 2006; Koley et al. 2017; Selesu et al. 2016).

**TABLE 3:** Recovery efficiencies reported in the literature for different microalgal flocculants applied at pilot‐scale.

Flocculant	Dose (mg/L)	Recovery (%)	pH	Microalga	References
FeCl_3_	200	80.2	7	*S. obliquus*	(Koley et al. 2017)
AlCl_3_	250	95	7	*S. obliquus*	(Koley et al. 2017)
NaOH		75	12	*S. obliquus*	(Koley et al. 2017)
		60	12	*C. vulgaris*	(Koley et al. 2017)
		80	10	*D. salina*	(Besson et al. 2019)
FO3801 cationic polymer		80	8–9	*C. vulgaris*	(Vu et al. 2021)
Magnafloc LT‐25	0.5	89	10–10.6	*T. pseudonana*	(Knuckey et al. 2006)
Chitosan	60	74	8.26	*C. vulgaris*	(Labeeuw et al. 2021)
	40	68.3	6.4–7.1	*T. striata*	(Figueira Garcia et al. 2024)
Microbial bio‐flocculant		> 98	3	*D. brasiliensis*	(Ndikubwimana et al. 2016)
Tanfloc	210	93–97	7.8	*Scenedesmus* sp.	(Selesu et al. 2016)

The efficiency of flocculants observed in a laboratory setting often differs significantly from that observed under outdoor mass culture conditions due to changes in the physicochemical properties of the culture medium, including variations in pH, dissolved oxygen, light intensity, and temperature (Table 4) (Figueira Garcia et al. 2024; Haver and Nayar 2017). These differences highlight the challenges of extrapolating laboratory results to an operational environment. It is widely accepted that flocculants used in pilot‐ and large‐scale systems should be inexpensive, non‐toxic, and effective (Grima et al. 2003; Xu et al. 2013). Scaling up flocculation from the laboratory to larger volumes usually results in reduced efficiency of metal cations due to less efficient mixing and limited pH control. However, chitosan has been found to be more effective in outdoor pilot‐scale systems than in laboratory‐scale experiments, likely due to its different charge neutralization mechanism, which involves adsorption to the microalgal cell surface (Figueira Garcia et al. 2024). Centrifugation of previously flocculated biomass comes with several challenges, such as a reduction in the dewatering capacity of the centrifuge compared to untreated cultures. This can be attributed to a few factors: the trapping of residual water between floc layers during centrifugation; alterations in the biochemical profile of the harvested biomass due to flocculation; interactions between flocculants and lipids or algal EPS; and cell damage during sedimentation, which can release intracellular compounds that alter floc density (Figueira Garcia et al. 2024; Labeeuw et al. 2021).

**TABLE 4:** Comparison between flocculation recoveries of selected flocculants in lab‐ and pilot‐scale.

	Recovery (%)		Dose (mg/L)		Microalga		References	
	Lab‐scale	Pilot‐scale	Lab‐scale	Pilot‐scale	Lab‐scale	Pilot‐scale	Lab‐scale	Pilot‐scale
AlCl_3_/Al_2_(SO_4_)_3_	> 99	95	152	250	*Chlorella* sp.	*S. obliquus*	(Sanyano et al. 2013)	(Koley et al. 2017)
FeCl_3_/Fe_2_(SO_4_)_3_	> 99	80.2	143	200	*Chlorella* sp.	*S. obliquus*	(Sanyano et al. 2013)	(Koley et al. 2017)
NaOH	90	60	/	/	*C. vulgaris*	*C. vulgaris*	(Vandamme et al. 2015)	(Koley et al. 2017)
Chitosan	99	74	120	60	*C. vulgaris*	*C. vulgaris*	(Rashid et al. 2013)	(Labeeuw et al. 2021)

Studies on the application of flocculants in pilot‐scale and large‐scale microalgae cultures are less common than those conducted at the laboratory scale (Besson et al. 2019; Figueira Garcia et al. 2024; Knuckey et al. 2006; Koley et al. 2017; Selesu et al. 2016). For example, the addition of 0.2 g/L FeCl_3_ and 0.25 g/L AlCl_3_ to 2 L cultures of *S. obliquus* resulted in recovery efficiencies of 80.2% and 95%, respectively (Koley et al. 2017). In pilot‐scale cultures (1000 L), increasing the pH to 12 without adding metal salts resulted in 75% and 60% flocculation efficiencies for *S. obliquus and C. vulgaris*, respectively (Koley et al. 2017). Similarly, pilot‐scale flocculation of *D. salina* using 0.02 M NaOH resulted in 80% recovery due to the precipitation of Mg(OH)_2_ from the seawater medium (Besson et al. 2019), with this type of acid/base flocculation requiring a magnesium concentration of [Mg^2+^] > 0.1 mM in the medium (Li et al. 2021). Pilot‐scale flocculation by adding 35 mg/g_DW_ of cationic polyacrylamide polymer FO3801 in 350 L cultures of *C. vulgaris* showed an increase in the flocculation efficiency that increased from 35% in the early exponential phase to 80% in the late stationary phase (Vu et al. 2021). This increase was explained by the zeta potential change (from the more negative to less negative) with culture aging, which implied faster flocculation in mature cultures (Vu et al. 2021). In a 130 L pilot‐scale flocculation of *T. pseudonana*, simultaneous pH increase (by adding 8 mM of NaOH) and the addition of 0.5 mg/L of nonionic polymer *Magnafloc LT‐25* resulted in a biomass recovery of 89%, with a concentration factor of 26 (Knuckey et al. 2006). A similar study reported the concentration factor of 14.29 for 0.1 g/L of AlCl_3_ or 0.1 g/L FeCl_3_ added to 100 L cultures of *T. striata* (Figueira Garcia et al. 2024). The higher efficiency of the synthetic polyelectrolyte was attributed to the increased sweeping power achieved through the combined effects of high pH and the industrial polyelectrolyte addition. The authors also successfully flocculated several microalgae species at larger volumes (10–1000 L), including *T. suecica* , *C. calcitrans* , *C. muelleri* , *Skeletonema* sp., *R. salina* , *A. septentrionalis* , *Nitzschia closterium* , and *C. muelleri* , with recovery efficiencies (≥ 85%), similar to those observed for *T. pseudonana* (Knuckey et al. 2006). In pilot‐scale studies (350 L), flocculation of the cyanobacterium *Synechocystis* sp., the freshwater *C. vulgaris* , and the marine *P. tricornutum* with chitosan showed that chitosan could not effectively flocculate *C. vulgaris* during the exponential phase, but achieved 74% recovery with 0.06 g/L chitosan during the stationary phase (Labeeuw et al. 2021). This is similar to the recovery rates of 68.3%–81.6% using 0.04–0.1 g/L of chitosan in 100 L cultures of three marine microalgal species (Figueira Garcia et al. 2024). In the latter study, the biomass recovery values for 100 L cultures were as follows: (1) 94.6% for *T. striata* with 0.08 g/L AlCl_3_, 88.4% for 0.1 g/L FeCl_3_, and 68.3% for 0.04 g/L chitosan; (2) 81.7% for *D. tertiolecta* with 0.1 g/L AlCl_3_, 87.9% for 0.2 g/L FeCl_3_, and 81.6% for 0.1 g/L chitosan; and (3) 89.6% for *C. sorokiniana* , with 0.1 g/L AlCl_3_, 98.6% for 0.2 g/L FeCl_3_, and 68.3% for 0.1 g/L chitosan (Figueira Garcia et al. 2024). Additionally, flocculation of large‐scale *D. brasiliensis* cultures using a microbial bio‐flocculant resulted in flocculation efficiencies exceeding 98% (Ndikubwimana et al. 2016). Pilot‐scale flocculation of 1000 L *Scenedesmus* sp. culture with 210 mg/L Tanfloc at pH = 7.8 resulted in recovery efficiencies of 93% to 97%, with only limited removal of TN and TOC (Selesu et al. 2016). The data summarized in Table 4 highlight a consistent trend: flocculation efficiencies are generally higher in laboratory‐scale systems than in pilot‐ or large‐scale applications, often achieved with lower flocculant dosages. This discrepancy is largely attributed to scale‐dependent operational limitations, including incomplete flocculant dissolution, uneven distribution, and suboptimal mixing in larger volumes. While several studies have demonstrated promising recovery rates at pilot scale—especially when using optimized pH conditions, synthetic polymers, or bio‐based flocculants—these data also emphasize the complexity of translating lab‐scale protocols to industrial settings. Future research should focus on improving flocculant delivery and mixing strategies at scale, as well as on the development of more effective and environmentally sustainable flocculants tailored for large‐volume microalgae harvesting.

## 3 Economic Assessment and Future Prospects

This section explores the costs associated with various flocculation agents, compares them to alternative harvesting methods, and discusses future prospects for improving the economic feasibility of this technology (Table 5). The cost of aluminum salts, a commonly used coagulant in microalgal harvesting, is approximately $250 per ton (Anthony and Sims 2013), while cationic starch is priced at $1000 per ton (Anthony and Sims 2013). When considering chitosan, a natural flocculant, the price for food‐grade chitosan is around 76.5 €/kg (Figueira Garcia et al. 2024). For flocculation pretreatment with chitosan, the cost per cubic meter of microalgal culture is calculated to be 3.15, 7.86, and 7.82 €/m^3^ for *T. striata* , *D. tertiolecta* , and *C. sorokiniana* , respectively (Figueira Garcia et al. 2024). The authors discuss that these values are significantly higher than the cost of a single‐step centrifugation process, which costs only 1.17 €/m^3^ for the same culture volume (Figueira Garcia et al. 2024). However, in another study conducted in the United States (US), flocculation efficiency improved with a concentration factor ranging from 20 to 50 when chitosan was added to *C. sorokiniana* at a concentration of 20 mg/L (Xu et al. 2013). Based on the commercial prices for chitosan in the US (20 USD/kg), the cost of the flocculant was estimated to be approximately 200 USD per ton of microalgae (Xu et al. 2013). In addition to chitosan, other common flocculants have been assessed for cost efficiency. For example, the cost of ferric chloride (FeCl_3_) was calculated at $130 per ton of algae, while aluminium chloride (AlCl_3_) costs around $65 per ton, and the synthetic cationic polymer “Zetag 8819” costs approximately $50 per ton (Udom et al. 2013). In Brazil, to achieve flocculation efficiency greater than 96% for 1000 L of *Scenedesmus* sp. culture, 25 g of chitosan and 433.25 mL of acetic acid are required. Assuming a biomass concentration of 0.5 g/L, this would result in a harvesting cost of $10.00 per kilogram of dry biomass, with $2.00 attributed to chitosan and $8.00 to acetic acid (Selesu et al. 2016). Alternatively, the use of Tanfloc, a commercial flocculant, can achieve greater than 96% flocculation efficiency for the same culture volume (1000 L) with 210 g of Tanfloc (costing $2.60/kg). In this case, the harvesting cost is reduced to $1.10/kg of dry biomass (Selesu et al. 2016). Further analysis using an optimized chitosan concentration of 46.10 mg/L, with an assumed chitosan cost of 20 USD/kg, suggests that the chitosan cost per kilogram of harvested biomass is approximately 1.87 USD (Zhu et al. 2025). Taking into account the energy consumption for the harvesting process, which is estimated at 0.2 kWh/m^3^ for green light irradiation and an electricity cost of 0.10 USD/kWh, the energy cost is approximately 0.43 USD per kilogram of harvested biomass (Zhu et al. 2025). Additionally, pH pretreatment for recovering 1 kg of *E. gracilis* dry biomass adds a small cost of around 0.05 USD. In total, the harvesting cost for *E. gracilis* is estimated at 2.35 USD per kilogram of dry biomass (Zhu et al. 2025).

**TABLE 5:** Price of the flocculation pretreatment, based on the data derived from reported findings in the available literature.

Flocculant	Price per ton of biomass	Year	Microalga	References
*Moringa oleifera*	1332.28 USD	2021	*C. vulgaris*	(Yang et al. 2021)
*Moringa oleifera*	877.96 USD	2021	*S. obliquus*	(Yang et al. 2021)
Chitosan	176.81 USD	2021	*C. vulgaris*	(Yang et al. 2021)
Chitosan	106.95 USD	2021	*S. obliquus*	(Yang et al. 2021)
Tanfloc	75.15 USD	2021	*C. vulgaris*	(Yang et al. 2021)
Tanfloc	50.25 USD	2021	*S. obliquus*	(Yang et al. 2021)
Cationic starch	85.35 USD	2021	*C. vulgaris*	(Yang et al. 2021)
Cationic starch	56.73 USD	2021	*S. obliquus*	(Yang et al. 2021)
AlCl_3_	599 USD^a^	2024	*D. tertiolecta*	(Figueira Garcia et al. 2024)
FeCl_3_	915 USD^a^	2024	*D. tertiolecta*	(Figueira Garcia et al. 2024)
Alum	3300 USD	2017	*S. obliquus*	(Koley et al. 2017)
FeCl_3_	2200 USD	2017	*S. obliquus*	(Koley et al. 2017)
Starch	2880 USD	2018	*Chlorella* sp.	(Min et al. 2022)
Chitosan	380 USD	2015	*C. reinhardtii*	(Min et al. 2022)
Cetrimonium bromide	6600 USD	2020	*Chlorella* sp.	(Min et al. 2022)
pH induced flocculation by NaOH	720 USD	2017	*S. obliquus*	(Koley et al. 2017)

^a^ These prices were converted from EUR to USD based on the current exchange rate on the 22nd of May 2025 (1 EUR = 1.13 USD).

When comparing these costs to industrial‐scale centrifugation, which is a commonly used method for algal biomass harvesting, the cost per cubic meter for centrifugation is significantly lower at 1.17 €/m^3^ (Figueira Garcia et al. 2024). This method, however, requires high energy input and can have operational complexities, making it less cost‐effective in large‐scale operations compared to flocculation methods when considering energy costs and operational efficiency.

## 4 Conclusions

Large‐scale microalgal biomass harvesting poses significant economic and operational challenges, representing a major bottleneck in the advancement of cost‐effective and sustainable microalgae‐based biotechnologies. While centrifugation is a reliable and effective separation method, its high energy demand, capital investment, and potential for cell damage limit its scalability. Flocculation has emerged as a promising pre‐concentration strategy, enabling more efficient biomass separation while reducing operational costs. Extensive laboratory studies have validated the effectiveness of various flocculants—including metal salts, natural polymers such as chitosan and cationic starch, and synthetic polyelectrolytes—across a wide range of microalgal strains. However, scaling up flocculation to pilot‐ and industrial‐scale applications presents significant challenges. Variability in flocculants performance due to fluctuating culture conditions, the need for precise dosage optimization to prevent over‐flocculation and biomass contamination, and the environmental impact of certain chemical flocculants complicate its implementation. Natural flocculants offer a more sustainable alternative, but their relatively high cost and limited scalability restrict widespread commercial adoption.

Pilot‐scale studies have shown that flocculation can reduce energy consumption and overall production costs, demonstrating its feasibility in real‐world operational environments. However, discrepancies between laboratory‐ and pilot‐scale efficiencies highlight the need for further research into process optimization, particularly in terms of flocculant selection, dosage control, and downstream processing compatibility. Economic assessments indicate that flocculation can significantly lower biomass separation costs compared to centrifugation alone; however, further advancements in cost‐effective, biodegradable flocculants, and seamless integration with existing harvesting systems are essential for widespread adoption.

Future research should prioritize standardizing pilot‐scale protocols, developing low‐cost and environmentally friendly flocculants, and integrating flocculation with other harvesting techniques to maximize efficiency. Addressing these challenges could establish flocculation as a pivotal technology for large‐scale microalgal biomass production, supporting the development of sustainable biofuels, bioproducts, and carbon capture solutions.

## Author Contributions

Conceptualization: A.S. and Z.G. Methodology: A.S. and Z.G. Investigation: Z.G. Writing – original draft preparation: V.R., A.S., and Z.G. Writing – review and editing: V.R., B.M., A.S., and Z.G. Supervision: B.M. Funding acquisition: B.M. All authors have read and agreed to the published version of the manuscript.

## Conflicts of Interest

The authors declare no conflicts of interest.

## Data Availability

The authors have nothing to report.
